# Applications of livestock monitoring devices and machine learning algorithms in animal production and reproduction: an overview

**DOI:** 10.1590/1984-3143-AR2023-0077

**Published:** 2023-08-28

**Authors:** Paula de Freitas Curti, Alana Selli, Diógenes Lodi Pinto, Alexandre Merlos-Ruiz, Julio Cesar de Carvalho Balieiro, Ricardo Vieira Ventura

**Affiliations:** 1 Departamento de Nutrição e Produção Animal, Faculdade de Medicina Veterinária e Zootecnia, Universidade de São Paulo, Pirassununga, SP, Brasil

**Keywords:** machine learning, computer vision, precision livestock farming, sensors

## Abstract

Some sectors of animal production and reproduction have shown great technological advances due to the development of research areas such as Precision Livestock Farming (PLF). PLF is an innovative approach that allows animals to be monitored, through the adoption of cutting-edge technologies that continuously collect real-time data by combining the use of sensors with advanced algorithms to provide decision tools for farmers. Artificial Intelligence (AI) is a field that merges computer science and large datasets to create expert systems that are able to generate predictions and classifications similarly to human intelligence. In a simplified manner, Machine Learning (ML) is a branch of AI, and can be considered as a broader field that encompasses Deep Learning (DL, a Neural Network formed by at least three layers), generating a hierarchy of subsets formed by AI, ML and DL, respectively. Both ML and DL provide innovative methods for analyzing data, especially beneficial for large datasets commonly found in livestock-related activities. These approaches enable the extraction of valuable insights to address issues related to behavior, health, reproduction, production, and the environment, facilitating informed decision-making. In order to create the referred technologies, studies generally go through five steps involving data processing: acquisition, transferring, storage, analysis and delivery of results. Although the data collection and analysis steps are usually thoroughly reported by the scientific community, a good execution of each step is essential to achieve good and credible results, which impacts the degree of acceptance of the proposed technologies in real life practical circumstances. In this context, the present work aims to describe an overview of the current implementations of ML/DL in livestock reproduction and production, as well to identify potential challenges and critical points in each of the five steps mentioned, which can affect results and application of AI techniques by farmers in practical situations.

## Introduction

Artificial Intelligence (AI) is a field that merges computer science (statistical models and algorithms) and large datasets to create intelligent systems that can perform tasks that normally require human intelligence, such as recognizing visual patterns, understanding languages, making decisions and processing speech (Sarker, 2022). In a simplified manner, Machine Learning (ML) is a branch of AI, and can be considered as a broader field that encompasses Deep Learning (DL - a Neural Network formed by at least three layers), generating a hierarchy of subsets formed by AI, ML and DL, respectively.

AI has been rapidly changing the ecosystem of different areas, from business operations to healthcare and livestock farming, due to the ability of providing predictions and insights through analysis of large datasets that result in information that can assist decision making (Rutten et al., 2013). With the world's population projected to reach 9.15 billion by 2050 (Alexandratos and Bruinsma, 2012), the demand for food is projected to increase continuously, making it crucial to find innovative solutions across different industries, such as in the animal and agriculture sectors, in a sustainable way.

In the animal production and reproduction field, the application of AI is not new (Rorie et al., 2002; Firk et al., 2002), but advancements in computing power, data capture and storage, in addition to cloud computing, have accelerated the pace of development of several methodologies in recent years. According to a recent review by Slob et al. (2021), the integration of AI in animal agriculture can be used to address multiple facets of dairy farm management, including animal health, milk production, and reproduction. Additionally, AI-based systems have also been studied to monitor animal behavior, detect early signs of disease (Rutten et al., 2013), collect phenotypes that are expensive or difficult to obtain, and identify environmental stressors that affect animal welfare (Neethirajan, 2020).

Besides the automation of tasks, AI can contribute by lowering production costs, enhancing productivity, reducing animal stress by minimizing human and animal interaction, and providing data-driven insights for better decision-making (Rutten et al., 2013). It also has the potential to be more precise than humans in certain situations, as this technology can process vast amounts of data quickly and efficiently, allowing the identification of patterns that are not easily detected and reducing the analysis subjectivity (Pinto et al., 2023), which are issues that could significantly affect the success of a project.

In order to implement a full cycle project using AI for farmers, there are some crucial steps, each with its particular attention points and challenges that must be taken into consideration: (1) data collection, (2) data transferring, (3) data storage, (4) data analysis, and (5) delivery of results to end-users. Throughout the articles reviewed herein, there are common pitfalls to be avoided and improvements that could be made in order to achieve better results in future studies. This review aims to explore the current applications of AI in livestock production and reproduction, in addition to highlighting key factors as part of the previously five steps mentioned, which can affect its implementation in practical circumstances.

## Technologies

The most widespread types of technologies used by farmers to attain information about their animals are wearable sensors and computer vision (CV) techniques, whose implementation varies according to the production system and the number of animals. One example of this phenomenon is in species such as sheep, goat, pig, poultry and fish, where the profit generated per animal does not allow the investment in individual sensors, only remaining the alternative approach of using the sensing methods applied to herd or flocks, where the unit of measurement is associated with the entire group (Halachmi et al., 2019).

In this context, the following sections will provide an overview of current technologies developed for livestock data collection.

### Sensors

According to the definition of Neethirajan (2020), a sensor is a device that measures or detects biological, chemical, physical or mechanical properties or its combinations. Through the adoption of these instruments, the data collected and recorded can be interpreted by a human or automatically analyzed by machine. The adoption of sensors in animal farming provides an opportunity to constantly monitor key animal and environmental biomarkers by continuously collecting data, which will posteriorly be explored using advanced AI and ML/DL algorithms to predict deviations or abnormalities for decision purposes (Neethirajan, 2020).

**Figure 1:**
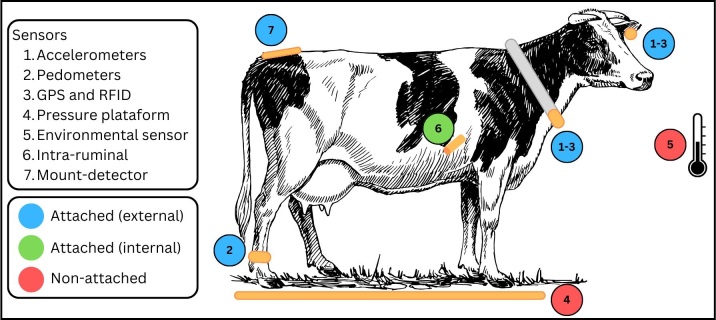
Overview of the positioning and types of sensors widely studied on precision livestock projects. **Figure cre**ated on Canva.

As reported by Rutten et al. (2013), sensors can be broadly classified in two main categories: attached and non-attached (Figure 1). The former refers to the application of devices outside of the body or inside it (physically attached to the animal). Conversely, non-attached sensors are positioned off-individual, considering that the animal will have information captured by passing/staying close to its installation. In-line and on-line sensors are subcategories of this second class. In-line sensors collect measurements in a continuous manner (without sampling) and are typically placed within the production line (e.g.: milk’s electrical conductivity, internally evaluated by the robotic milking systems). On-line sensors, on the other hand, are not integrated in the main flow and are designed to conduct automatic periodic sampling that is subsequently analyzed (e.g. Somatic Cell Count) (Rutten et al., 2013; Bowler et al., 2020).

One important factor to take into consideration regarding the effectiveness of the sensor data collection is, according to Halachmi et al. (2019), the amount of false-positive and false-negative alerts provided by the system, which is closely associated to the degree of correlation between the biomarker measured by the sensor and the final target information the farmer is trying to obtain. Also, distinct sensors function in different ways, and not all sensors can offer meaningful insights regarding a particular biological or environmental event.

- Accelerometers: through the measurement of symmetry, differences of acceleration and positioning of the animal’s body members (Halachmi et al., 2019), a farmer is able to have insights of the animal’s behavior, health, nutrition and reproductive status (Morrone et al., 2022).
- Radio Frequency Identification (RFID): is used in many ways in livestock production systems to identify, track and manage animals, as it allows correct assignment of data to a specific animal and rapid transfer to a central repository with minimal error (Berry, 2023). There are different types of radio frequency identification systems (active, passive and hybrid tags), and the main differences between them lie in the range of the reading and size of the sensor, as active ones can receive signals from a greater distance but are bigger in size since it contains more electrical parts (Mishra and Sharma, 2023).
- Force and pressure platforms: allow assessment of force and pressure that an animal applies to the platforms. It is usually used to analyze gait patterns, as it allows insights of the forces exerted when the animal walks over it (dynamic assessment) or stands on the platforms (static assessment) (Nejati et al., 2023).
- Environmental sensors: used to measure temperature, gas production, humidity, and other parameters (Neethirajan, 2020), which is important for animal comfort, disease, greenhouse gas emission and production management (Gray et al., 2017; Tullo et al., 2019).
- Other types

Another commercially available technology in this domain is wireless intra-ruminal sensors, exemplified by the SmaXtec system (https://smaxtec.com/en/), capable of capturing data on rumen motility, pH, and body temperature. This technology can offer insights for early identification of conditions such as ruminal acidosis (Schori and Münger, 2022).

Another research area that has been widely adopted in recent years to obtain and analyze data through images and videos, is Computer Vision (CV), which will be described in the following section.

### Computer vision techniques

CV can be described as a branch of the AI field that allows a machine to extract relevant information from physical objects through images (Ballard and Brown, 1982), which may comprise still pictures or individual frames extracted from a video. In the context of livestock production systems, it is observed that CV adoption is expanding due to the fact that it can extract real time, non-invasive and accurate animal-level information (Oliveira et al., 2021).

In CV, the employed camera directly affects the type and quality of data being collected. For instance, RGB cameras can identify changes in color and movement, however precision is lost when monitoring multiple animals at once, as each individual occupies fewer pixels in the video frame (Wu et al., 2023). Infrared thermography cameras have the ability to measure and image the heat radiation in the infrared part of the light spectrum emitted by any object, so it can offer insights regarding temperature deviations on an animal's body (Kunc et al., 2007). Finally, 3D cameras are able to reconstruct the animal’s anatomy (totally or partially), which is useful to analyze anatomical asymmetries (Abdul Jabbar et al., 2017). These tridimensional cameras are also important to evaluate animal health and production by indirectly measuring key parameters, such as weight and body condition (Qiao et al., 2021), commonly evaluated during daily activities by farmers.

## Applications

### Animal reproduction

In the animal reproduction field, many advancements have been made through the implementation of ML techniques in various tasks. Estrus detection was one of the major subjects studied, since the rates of detection have been reported to be falling for a few decades (Mottram, 2016). Rutten et al. (2013) identified 41 publications dedicated to automated detection of estrus, including activity detection (pedometers, activity meters, and 3-dimensional accelerometers), progesterone level in milk (biosensors and immunostrips), mounting behavior (sensor and video camera), vocalization (microphone), and body temperature (temperature transducer and bolus inserted in the cow’s reticulum). Besides the technologies described, Göncü and Koluman (2019) also reported the use of pressure activated mount detectors, temperature measurement and radio telemetric transmission. Accelerometers and pedometers were the most studied sensors according to these studies (Rutten et al., 2013; Göncü and Koluman, 2019).

Regarding CV applied to estrus detection, Noe et al. (2022) proposed a CV-based system using DL methodologies in order to identify cattle mating posture, as it is a specific behavior expressed during estrus, with 95.5% accuracy rate. Additionally, Aungier et al. (2015), when designing activity clusters through accelerometer-based data, to investigate their connection with endocrine profiles and ovulation timing in Holstein-Friesian cows, found moderate positive correlation (r = 0.53) between activity clusters and estrous-related behavior. However, no significant relationship was observed between activity clusters and the cow's endocrine profiles. In an attempt to identify risk factors associated with measurements of estrus expression and parturition/artificial insemination, a study employing various sensors (such as a neck-mounted accelerometer and a leg-mounted pedometer) revealed that a greater peak of physical activity index and duration of each estrus episode, as body condition score increased in their evaluation. (Madureira et al., 2015)

In another review on estrus detection, Mottram (2016) described the importance of combining different measures for this end, such as performing a multivariate analysis or observing changes in patterns of data from multiple sources. However, the author noted that including variables does not necessarily improve the results, and that both adding extra sensors and fusing various sets of data may provide new challenges.

Concerning sperm viability, while seeking to improve some setbacks encountered in Computer Assisted Sperm Analysis (CASA) bull sperm motility evaluation, Hidayatullah et al. (2021) proposed a ML model based on three parameters used in CASA (curvilinear velocity, straight line velocity and linearity) using a 500x magnification, obtaining 92.08% mean accuracy. Also, Keller and Kerns (2022) applied techniques developed in the human cancer research field in order to create an AI tool based in DL analysis of boar sperm that allows the evaluation of acrosome integrity solely on brightfield images, which eliminates the need for fluorescent biomarkers, facilitates the identification of boars with low conception rates and enables a reduction of the sperm count per insemination dose, so higher genetic indexing boars can be used more effectively in the herd (Keller and Kerns, 2022).

On the subject of embryo morphology and viability scoring, many studies adopted CV and ML/DL techniques to assess the problem of early pregnancy loss due to embryonic incompetence for survival. Matos et al. (2014) developed a software able to automatically assign scores based on the structural quality of mouse blastocyst, acquiring a 95% accuracy rate. Likewise, Rocha et al. (2017a) carried out the same procedures to bovine blastocysts, attaining a 76.4% accuracy rate. In both cases, the models were successful due to a previous thorough image pre-processing process (Rocha et al., 2017b). More recently, in human embryology, Berntsen et al. (2022) used a large dataset of embryo images (n=115,832) and developed a robust and fully automated DL model, able to select viable embryos for implantation or cryopreservation, which had superior outcomes compared to other techniques.

Considering the challenges faced to achieve higher genetic gains, Berry (2023) states that one of the greater benefits of the improvements made in sensing systems and data analysis is to provide novel approaches to measure traits that are known to be challenging to quantify. One example that illustrates this is the research conducted by Rabaglino et al. (2023), where the authors identified genes linked to embryonic competency and viability, by merging transcriptomic data and applying validation techniques using ML algorithms.

### Animal production

Many efforts have been dedicated to design solutions for challenges faced in specific practical situations related to animal production, including animal nutrition, health, overall production and food quality.

Nutrition: according to Kyriazakis (2023), the term Smart Nutrition describes the method of feeding animals with the help of smart devices (such as cameras and sensors), that through communication with other devices or networks and/or the use of data analytics and AI, are able to capture information automatically and generate outputs that can be used for the management of livestock systems. The use of these technologies are already described in cattle (Gonzáles et al., 2023; Kyriazakis, 2023), poultry (Zuidhof et al., 2023), pigs (Brossard et al., 2023), fish (Fore et al., 2023) and other animals, offering solutions that encompass automated individual feed intake quantification (Bezen et al., 2020), feeding behavior (Bloch et al., 2023), rumination pattern tracking (Kliemann et al., 2023), pasture management and biomass calculation (Castro et al., 2020), among others.
Health and behavior: many studies proposed AI and sensor-based alternatives to identify, monitor and control health-related features, such as early mastitis identification in dairy cows (Wang et al., 2022), gait analysis and lameness detection (Zhao et al., 2018; Nejati et al., 2023), respiratory pattern (Wu et al., 2020, 2023), diarrhea and respiratory disease in calves (Ghaffari et al., 2022), body condition score (Huang et al., 2019), body temperature measurement (Giro et al., 2019; Barreto et al., 2022), and behavior monitoring (Li et al., 2019; Watanabe et al., 2021; Gong et al., 2022).
Production: some examples of technological advancements made in the animal production field are illustrated in the dairy and meat industries. Liseune et al. (2021) were able to successfully create a DL model able to predict a cow’s lactation curve based on the historical sequence of milk yield, as well as reproduction and health issues that occurred in the previous cycle. In the meat industry, developments have been made in the prediction of carcass composition while the animal is still alive through sensing methodologies (computed tomography, dual-energy X-Ray absorptiometry and CV) (Leighton et al., 2022).
Food quality: in a review regarding sensing technologies used to evaluate carcass composition and quality of meat and fat, Leighton et al. (2022) pointed out that CV systems have many applications in this area of expertise, as seen in Pinto et al. (2023), where a model capable of classifying different intramuscular fat patterns in the ribeye area was trained, providing an automatic assessment to meat marbling scoring. Moreover, in order to guarantee milk quality and safety, Lima et al. (2022) developed a high performance process to detect milk adulteration with cheese whey through Fourier-transform infrared spectroscopy associated with DL techniques.

## Challenges regarding the implementation of precision livestock farming techniques

One of the challenges faced in recent years is a result of improvements made in sensing devices and the expansion of the internet of things. Massive production of raw data is ongoing (Morrone et al., 2022) and expected to persist in the near future, which needs to be stored locally for further post-processing or transmitted immediately to another physical or virtual location, evaluated and reported back for management decisions. Raw data may also be evaluated (by processes such as feature extraction) and deleted afterwards. In this scenario, the processed data (containing only relevant information) becomes the set of files that must be stored and kept for further analysis.

**Figure 2:**
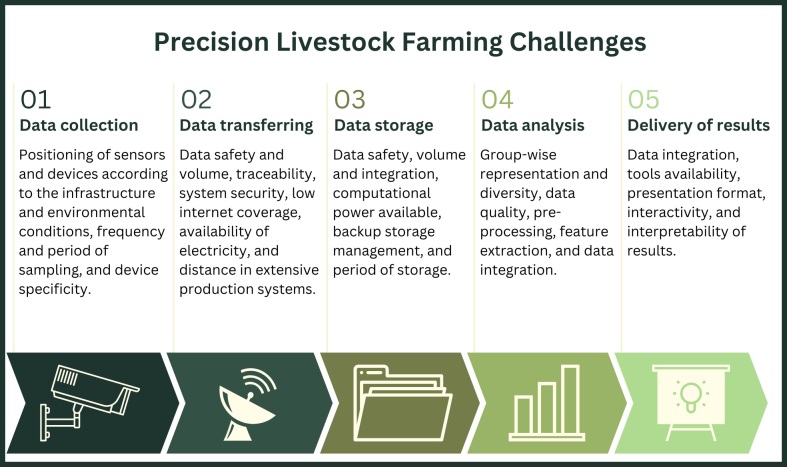
Major challenges faced in the execution of a full-cycle precision livestock farming project, step-by-step. Figure created on Canva.

In this context, in order to design a full cycle project aiming maximum automatization and autonomy, this review will address significant aspects that need to be taken into consideration when designing a PLF project, dividing it in five crucial steps: (1) data collection, (2) data transferring, (3) data storage, (4) data analysis, and (5) delivery of results to end-users. Figure 2 presents a comprehensive overview of each step, along with some of the challenges it encompasses.

Most studies are focused on the sensor technologies and data analysis aspects, which are key components of any PLF project. However, other steps are often overlooked or not fully described. One example are the obstacles encountered during data collection and transferring in remote regions, such as larger areas that characterize the majority of beef cattle system production in Brazil. Another example is on the final stage, where the data must be fully analyzed and integrated to generate decision tools for the producers. Thus, in order to develop a full cycle project, it is recommended to give significant importance to each of the following steps outlined below.

### Data collection

In a review over the application and prospective discussion of ML for the management of dairy farms, Cockburn (2020) described that ML algorithms have become common tools to predict data and other applications in dairy research. However, most of the tested algorithms could not be considered reliable for practical implementation due to unsatisfactory sensitivity and/or specificity performance. The author attributed this to poor data quality in the training sets, a problem directly impacted by the data collection procedure.

Some other technical challenges during data collection involve sensor positioning, sampling frequency (Neethirajan, 2020) and animal-related characteristics, such as breed, age, and production system, which are often generalized/extrapolated (Kliemann et al., 2023). According to Neethirajan (2020), these factors not only affect the algorithm performance, but also the feasibility and scalability of the proposed solution.

The challenge described above was observed in the research conducted by Kliemann et al. (2023). In their study, they validated the usage of the SCR Heatime HR System sensor on 180 kg body weight dairy Gyr heifers, for analyzing rumination patterns and comparing them to behavioral parameters in different production systems. However, their findings revealed that the sensor tended to overestimate data related to rumination. As a result, it was concluded that the sensor is not suitable for accurately measuring this parameter in a tie-stall and loose-housing system, considering this particular breed at the evaluated age.

One proposed solution by Cockburn (2020) is acquiring data from multiple farms and covering the measurements over longer periods, which could improve the accuracy of predictions. However, information integration and accessibility of public data continue to pose significant challenges to this day.

### Data transferring

Data transmission is a critical process responsible for transferring the input obtained from sensors and devices to servers and applications, in a safe manner. Also, it is one of the major bottlenecks in PLF projects, especially in scenarios associated with remote environments comprising large areas, due to low internet coverage, and in many cases, with no available electricity in the region in which the metrics will be captured in real time. The data transfer success is directly associated with the type and variety of data being collected (logs obtained via sensors, images, videos or sound, for example), as well as the frequency of collection and number of transmission events.

Production systems, such as beef cattle production in its extensive form, that explore large territorial extensions (characteristic of countries like Brazil), are quite important for maximizing the production in a low costs manner. Such environments make it difficult to collect massive data from specific types, such as images, videos or audio, specially by the difficulty of positioning cameras and other devices along the environment where the animals are raised. According to Akhigbe et al. (2021), additional efforts are required in order to find a balance among data rate, communication coverage and energy consumption during the data transmission process, and several researches have proposed different alternatives to transmit data between two locations that are distantly located (Sasloglou et al., 2009; Jegan et al., 2022; Klaina et al., 2022).

Discussing the application of fourth (4G) and fifth (5G) wireless network technologies in a review involving the Industry 4.0 and PLF, Morrone et al. (2022) points out that both network approaches are not widely implemented in certain areas, which makes new PLF projects dependent on the usage of satellite services or implementation of other network types. In order to assess this problem, one proposed approach is the use of LPWAN (Low-Power Wide Area Network) devices, as they associate wireless technology with low-power consumption, at the same time that allows long-range coverage (Buurman et al., 2020). Unlike other wireless network options, such as Wi-Fi, Bluetooth and the mobile “G” options (from 3 to 5), developed to offer high-speed over short distances at the cost of having a higher power consumption (Pasolini, 2022), the LPWAN model is able to cover large areas such as pastures, and thus it is frequently adopted for projects that connect devices designed to generate limited volume of data associated with a low transfer rate over longer distances. LoRa (Long Range) is one example of the LPWAN class that uses a chirp spread spectrum modulation and has been widely used in rural remote areas in association with photovoltaic panels as the power source.

It is important to note that all the data transferred must find a proper way through networks and protocols. However, this task demands detailed information about the data itself, such as type, origin, size and destination, which are required to form a package that must be transferred in small blocks of information. As illustrated by Zhang et al. (2018), the collected information is structured as a data unit packet to provide the metadata needed to identify the origin of the data and how it was composed. They have created a specific protocol to encapsulate all this information about the data and to create a unit packet to be transmitted, as it is essential to trace back the information and also to check if the data is corrupted during transmission. Such protocol improves the security of the system, since it becomes possible to validate the authenticity of the data transferred.

### Data storage

As discussed before, a large volume of data is needed to implement ML and DL algorithms in order to solve PLF problems. As reported by Neethirajan (2020), different datasets from distinct biological origins, such as weather and air quality data, sound from animals, and behavior data, are required to apply mechanistic models in animal farming. This data type diversity must be stored in a database schema that allows multiple data types and does not require fixed forms, as is observed for scenarios when media (image and video) is captured.

Souza et al. (2022) investigated the adoption of a non-relational (MongoDB) database solution to load both unstructured data (different sets of novel phenotypes) and data with predefined patterns (genomic information using different data formats). The authors concluded that data conversion was a crucial step associated with high computational cost, required to manage different data types before loading into the database structure. These authors indicated that the increasing number of applications that generate or handle audio and videos, required the development of appropriate tools for handling the conversion of these suggested data types. Sampaio et al. (2018) carried out research to explore the Video7 architecture for storing and retrieving media streaming files into non-relational databases.

A main question regarding data storage and PLF projects is associated with the temporal effect of data collection (monthly, weekly, daily, hourly or every second), and for how long it should be stored after being collected, in addition to the problem of keeping raw data versus only the results of feature extraction. In certain situations, one minute video recording using a RGB-D camera in a max resolution for RGB and Depth modes at the same time, considering only 6 frames per second, will generate 2.5 GBs of raw data, which suggests that multiple cameras collecting data 24/7 will require a special protocol for data collection, in order to avoid the raw data retrieval.

### Analysis

A typical pipeline, considering specifically the analysis step and the adoption of ML and DL methods to solve biological problems, consists in: i) gathering data already collected and previously organized in a database solution; ii) preprocessing for cleaning, transformation and normalization purposes; iii) feature extraction and/or creation of new attributes (depending on the application and via adoption of DL algorithms, this step is not required); and iv) defining the models and algorithms to be investigated, with subsequent training/evaluation methods prior to deployment.

Feature extraction aims at selecting the most relevant features in problems such as classification and is an important step that involves selection and transformation of raw data into meaningful information (Pinto et al., 2023). An appropriate feature extraction pipeline allows data reduction (in terms of number of variables to be analyzed), in addition to identifying potential crucial features or attributes that are associated with the target variable. Thus, the computation time required to perform the analysis is reduced when compared with the same model being applied on the full dataset, which can posteriorly affect the final time for training and for the deployment steps (Rosales-Pérez, 2022). Depending on the problem and also considering the hardware infrastructure and architecture for transmitting data, feature extraction can be applied on the raw data before transferring in order to optimize the subsequent processes, such as data transferring, storage and analysis. This step depends directly on the biological nature of the problem, which algorithms are available to perform such a task as a pre-processing approach, and if the computation environment at the data collection stage is adequate economically.

In a comprehensive analysis made by Oliveira et al. (2021) regarding DL algorithms used in CV designed to livestock, the authors revealed that YOLO and Faster R-CNN were the primary algorithms used for object detection, while Mask R-CNN, U-Net and Deeplab models, were employed for object segmentation. Furthermore, the research found a diversity of DL architectures across studies, with Resnet, Xcpetion, VGG16, Inception and Darknet being among the most commonly featured.

Slob et al. (2021), in a review covering the application of ML to improve dairy farm management, after examining studies from several different research teams, reported that the main challenges were associated with unbalanced and small datasets, feature selection, overfitting/estimating, and the parameter tuning process. The same authors revealed that twenty-three different algorithms were identified across the reviewed publications, where the tree-based methods were the most used overall, followed by neural networks and regressions, respectively. Sensors were the data source with higher reference during the feature engineering step and there was a new trend in terms of algorithms adoption, with a broader adoption of neural networks algorithms in the most recent years, compared with the tree-based ones.

The strategy of incorporating pre-trained weights on deep learning usually presents the benefit of fastening the training process and boosting the network convergence, in particular when using small datasets. However, an important gap in the literature is the lack of information regarding which layers of the network were re-trained, or whether the entire network were, despite the fact that the DL architecture is frequently known and well documented (Oliveira et al., 2021).

### Delivery of results

Another important step, yet often overlooked, is the delivery of results. The ultimate goal of applying AI to production systems is to provide producers with a tool that will enable them to make informed decisions. This can only be achieved if the end user, the farmer, is able to interpret the information that is being presented, and use it in the decision making process.

As discussed, since data often comes from multiple sensors, cameras, and analyses, in order to make it presentable it is paramount to integrate it in the previous steps (data storage and data analysis) and also during the delivery of results. According to Cockburn (2020), the current use of data in dairy farms is not at its full potential, as many proposed solutions focus on one variable/characteristic instead of combining information to make them more effective. Furthermore, the author emphasizes that this has a significant influence on the acceptance of the technology by the end-user, as they consider that the benefits offered do not outweigh the cost associated with the sensing device.

Data integration is important because each gadget and analysis has its own limitations and may be subject to eventualities, in which case the complementary resources are able to contribute for a more accurate prediction/monitoring or for the compensation of eventual failures. Moreover, a more complete overview of the system may facilitate the delivery of technical recommendations that could be more easily interpreted and adopted by the end-user, which is often lacking in existing applications.

As described by Li et al. (2018), a multitude of integrative analyses can be accomplished through ML techniques, including feature concatenation, Bayesian models, multiple kernel learning, among others. Cabrera et al. (2020) proposed a system which would implement ML/DL algorithms on data commonly collected in dairy farms, integrating information from feeding schedules, herd management systems, and the milking parlor or automatic milking system software, thus allowing for improved management decisions (Cockburn, 2020). According to Li et al. (2018), Python language presents as a promising environment for executing integrative models, due to its applicability in large datasets and the development of extensive packages. Additionally, Python is known for its user-friendly nature, enabling a wide range of individuals to utilize it,allowing the direct contribution in the development stage of collaborators from different fields of study (e.g. veterinarians, agronomists, data scientists, engineers, and others), which is an essential attribute for PLF projects. (Norton et al., 2019; Morrone et al., 2022).

Another promising solution for this problem lies in the use of Business Intelligence tools. These instruments are largely adopted by businesses, since they provide a base for data-driven decision making by managers and leaders (Foley and Guillemette, 2010). Softwares such as Tableau and Power BI are interesting tools that enable data integration from many sources and in distinct formats. For instance, Power BI allows the user to connect to flat files, SQL databases, Azure cloud, and even web platforms such as Facebook, Google Analytics, and Salesforce objects (Seminger et al., 2023).

Besides integrating data, Business Intelligence tools enable the generation of Dashboards, pages that gather graphics and allow the user to apply filters and select data across multiple databases, updating the entire screen. This can be an attractive way of presenting results, in addition to making it easier for the users to interpret and extract valuable information from the data. For instance, Selli et al. (2021) made use of Tableau for integrating results from several genomic analyses across 17 worldwide sheep populations, and identified trends that otherwise would require more graphics and a longer time to analyze.

Finally, the delivery step is where all of the knowledge developed by the application is handled to the producer. The intermediate steps will require a lot of computation, as opposed to the collection step, and interactions in these steps will usually make use of a real-time active database (Akhigbe et al., 2021).

## Conclusion

The utilization of sensors and other data collection techniques, such as CV, serves as a viable alternative to obtain quantitative information from animals while reducing data collection costs, as they enable data acquisition over an extended period, offer the potential for automating processes on the farm and the possibility of making data-based informed decisions.

In the reviews addressed in our work, it is clear that PLF methods are currently under development to assess various challenges existing in the animal production and reproduction fields, and there is a future trend towards an expansion of the usage of such techniques. However, its adoption by end-users still is not at its full potential. In order to address this issue and optimize the practical implementation of new PLF projects, this work targeted important aspects to be taken into consideration during the different steps involved in the creation of a full cycle project: data collection, transferring, storage, analysis and delivery of results.

## References

[B001] Abdul Jabbar K, Hansen MF, Smith ML, Smith LN (2017). Early and non-intrusive lameness detection in dairy cows using 3-dimensional video. Biosyst Eng.

[B002] Akhigbe BI, Munir K, Akinade O, Akanbi L, Oyedele LO (2021). IoT technologies for livestock management: a review of present status, opportunities, and future trends. BDCC.

[B003] Alexandratos N, Bruinsma J (2012). World agriculture towards 2030/2050: the 2012 revision.

[B004] Aungier SPM, Roche JF, Duffy P, Scully S, Crowe MA (2015). The relationship between activity clusters detected by an automatic activity monitor and endocrine changes during the periestrous period in lactating dairy cows. J Dairy Sci.

[B005] Ballard DH, Brown CM (1982). Computer vision..

[B006] Barreto ADN, Barioni W, Pezzopane JRM, Bernardi ACDC, Pedroso ADF, Marcondes CR (2022). Thermal comfort and behavior of beef cattle in pasture-based systems monitored by visual observation and electronic device. Appl Anim Behav Sci.

[B007] Berntsen J, Rimestad J, Lassen JT, Tran D, Kragh MF (2022). Robust and generalizable embryo selection based on artificial intelligence and time-lapse image sequences. PLoS One.

[B008] Berry DP, Kyriazakis I (2023). Smart livestock nutrition..

[B009] Bezen R, Edan Y, Halachmi I (2020). Computer vision system for measuring individual cow feed intake using RGB-D camera and deep learning algorithms. Comput Electron Agric.

[B010] Bloch V, Frondelius L, Arcidiacono C, Mancino M, Pastell M (2023). Development and analysis of a CNN- and transfer-learning-based classification model for automated dairy cow feeding behavior recognition from accelerometer data. Sensors (Basel).

[B011] Bowler AL, Bakalis S, Watson NJ (2020). A review of in-line and on-line measurement techniques to monitor industrial mixing processes. Chem Eng Res Des.

[B012] Brossard L, van Milgen J, Dourmad J-Y, Gaillard C, Kyriazakis I (2023). Smart livestock nutrition..

[B013] Buurman B, Kamruzzaman J, Karmakar G, Islam S (2020). Low-power wide-area networks: design goals, architecture, suitability to use cases and research challenges. IEEE Access.

[B014] Cabrera VE, Barrientos-Blanco JA, Delgado H, Fadul-Pacheco L (2020). Symposium review: real-time continuous decision making using big data on dairy farms. J Dairy Sci.

[B015] Castro W, Marcato J, Polidoro C, Osco LP, Gonçalves W, Rodrigues L (2020). Deep learning applied to phenotyping of biomass in forages with UAV-based RGB imagery. Sensors (Basel).

[B016] Cockburn M (2020). Review: Application and prospective discussion of machine learning for the management of dairy farms. Animals (Basel).

[B017] Firk R, Stamer E, Junge W, Krieter J (2002). Automation of oestrus detection in dairy cows: a review. Livest Prod Sci.

[B018] Foley É, Guillemette MG (2010). What is business intelligence?. Int J Bus Intell Res.

[B019] Føre M, Alver MO, Frank K, Alfredsen JA, Kyriazakis I (2023). Smart livestock nutrition..

[B020] Ghaffari MH, Monneret A, Hammon HM, Post C, Müller U, Frieten D, Gerbert C, Dusel G, Koch C (2022). Deep convolutional neural networks for the detection of diarrhea and respiratory disease in preweaning dairy calves using data from automated milk feeders. J Dairy Sci.

[B021] Giro A, Bernardi ACDC, Barioni W, Lemes AP, Botta D, Romanello N (2019). Application of microchip and infrared thermography for monitoring body temperature of beef cattle kept on pasture. J Therm Biol.

[B022] Göncü S, Koluman N (2019). The sensor technologies for more efficient cow reproduction systems. MOJES.

[B023] Gong C, Zhang Y, Wei Y, Du X, Su L, Weng Z (2022). Multicow pose estimation based on keypoint extraction. PLoS One.

[B024] González LA, Imaz JA, Chang-Fung-Martel J, Kyriazakis I (2023). Smart livestock nutrition..

[B025] Gray J, Banhazi TM, Kist AA (2017). Wireless data management system for environmental monitoring in livestock buildings. Inf Process Agric.

[B026] Halachmi I, Guarino M, Bewley J, Pastell M (2019). Smart Animal Agriculture: application of real-time sensors to improve animal well-being and production. Annu Rev Anim Biosci.

[B027] Hidayatullah P, Mengko TLER, Munir R, Barlian A (2021). Bull sperm tracking and machine learning-based motility classification. IEEE Access.

[B028] Huang X, Hu Z, Wang X, Yang X, Zhang J, Shi D (2019). An improved single shot multibox detector method applied in body condition score for dairy cows. Animals (Basel).

[B029] Jegan G, Sheeba IR, Priya PK, Joany RM, Vino T (2022). Cattle tracking system architecture using LORA..

[B030] Keller A, Kerns K (2022). Deep learning, artificial intelligence methods to predict boar sperm acrosome health. Anim Reprod Sci.

[B031] Klaina H, Guembe IP, Lopez-Iturri P, Campo-Bescós MÁ, Azpilicueta L, Aghzout O (2022). Analysis of low power wide area network wireless technologies in smart agriculture for large-scale farm monitoring and tractor communications. Measurement.

[B032] Kliemann RD, Fernandes SR, Campos MM, Tomich TR, Pereira LGR, Neto AFG (2023). Sensor validation to record rumination and analysis of behavioral parameters of dairy Gyr heifers in feedlot systems. Trop Anim Health Prod.

[B033] Kunc P, Knízková I, Prikryl M, Maloun J (2007). Infrared thermography as a tool to study the milking process: a review article. Agric Trop Subtrop.

[B034] Kyriazakis I, Kyriazakis I (2023). Smart livestock nutrition..

[B035] Leighton PLA, Segura J, Lam S, Marcoux M, Wei X, Lopez-Campos O, Soladoye P, Dugan MER, Juarez M, Prieto N (2022). Prediction of carcass composition and meat and fat quality using sensing technologies: a review. Meat and Muscle Biology..

[B036] Li X, Cai C, Zhang R, Ju L, He J (2019). Deep cascaded convolutional models for cattle pose estimation. Comput Electron Agric.

[B037] Li Y, Wu FX, Ngom A (2018). A review on machine learning principles for multi-view biological data integration. Brief Bioinform.

[B038] Lima JS, Ribeiro DCSZ, Neto HA, Campos SVA, Leite MO, Fortini MEDR, de Carvalho BPM, Almeida MVO, Fonseca LM (2022). A machine learning proposal method to detect milk tainted with cheese whey. J Dairy Sci.

[B039] Liseune A, Salamone M, Van den Poel D, van Ranst B, Hostens M (2021). Predicting the milk yield curve of dairy cows in the subsequent lactation period using deep learning. Comput Electron Agric.

[B040] Madureira AML, Silper BF, Burnett TA, Polsky L, Cruppe LH, Veira DM, Vasconcelos JL, Cerri RL (2015). Factors affecting expression of estrus measured by activity monitors and conception risk of lactating dairy cows. J Dairy Sci.

[B041] Matos FD, Rocha JC, Nogueira MFG (2014). A method using artificial neural networks to morphologically assess mouse blastocyst quality. J Anim Sci Technol.

[B042] Mishra S, Sharma SK (2023). Advanced contribution of IoT in agricultural production for the development of smart livestock environments. Internet of Things (Netherlands).

[B043] Morrone S, Dimauro C, Gambella F, Cappai MG (2022). Industry 4.0 and Precision Livestock Farming (PLF): an up to date overview across animal productions. Sensors (Basel).

[B044] Mottram T (2016). Animal board invited review: precision livestock farming for dairy cows with a focus on oestrus detection. Animal.

[B045] Neethirajan S (2020). The role of sensors, big data and machine learning in modern animal farming. Sens Biosensing Res.

[B046] Nejati A, Bradtmueller A, Shepley E, Vasseur E (2023). Technology applications in bovine gait analysis: a scoping review. PLoS One.

[B047] Noe SM, Zin TT, Tin P, Kobayashi I (2022). Automatic detection and tracking of mounting behavior in cattle using a deep learning-based instance segmentation model. Int J Innov Comput, Inf Control.

[B048] Norton T, Chen C, Larsen MLV, Berckmans D (2019). Review: Precision livestock farming: building ‘digital representations’ to bring the animals closer to the farmer. Animal.

[B049] Oliveira DAB, Pereira LGR, Bresolin T, Ferreira REP, Dorea JRR (2021). A review of deep learning algorithms for computer vision systems in livestock. Livest Sci.

[B050] Pasolini G (2022). On the LoRa chirp spread spectrum modulation: signal properties and their impact on transmitter and receiver architectures. IEEE Trans Wirel Commun.

[B051] Pinto DL, Selli A, Tulpan D, Andrietta LT, Garbossa PLM, Voort GV, Munro J, McMorris M, Alves AAC, Carvalheiro R, Poleti MD, Balieiro JCC, Ventura RV (2023). Image feature extraction via local binary patterns for marbling score classification in beef cattle using tree-based algorithms. Livest Sci.

[B052] Qiao Y, Kong H, Clark C, Lomax S, Su D, Eiffert S (2021). Intelligent perception for cattle monitoring: a review for cattle identification, body condition score evaluation, and weight estimation. Comput Electron Agric.

[B053] Rabaglino MB, Salilew‐Wondim D, Zolini A, Tesfaye D, Hoelker M, Lonergan P, Hansen PJ (2023). Machine‐learning methods applied to integrated transcriptomic data from bovine blastocysts and elongating conceptuses to identify genes predictive of embryonic competence. FASEB J.

[B054] Rocha JC, Passalia FJ, Matos FD, Takahashi MB, Ciniciato DDS, Maserati MP, Alves MF, Almeida TG, Cardoso BL, Basso AC, Nogueira MFG (2017). A method based on artificial intelligence to fully automatize the evaluation of bovine blastocyst images. Sci Rep.

[B055] Rocha JC, Passalia FJ, Matos FD, Takahashi MB, Maserati MP, Alves MF, de Almeida TG, Cardoso BL, Basso AC, Nogueira MFG (2017). Automatized image processing of bovine blastocysts produced in vitro for quantitative variable determination. Sci Data.

[B056] Rorie RW, Bilby TR, Lester TD (2002). Application of electronic estrus detection technologies to reproductive management of cattle. Theriogenology.

[B057] Rosales-Pérez A, Torres Garcia AA (2022). Biosignal processing and classification using computational learning and intelligence..

[B058] Rutten CJ, Velthuis AGJ, Steeneveld W, Hogeveen H (2013). Invited review: sensors to support health management on dairy farms. J Dairy Sci.

[B059] Sampaio VSOL, De Macedo DDJ, Britto A (2018). Video7: An Architecture for Storage and Recovery of Streaming Audio and Video in NoSQL Database..

[B060] Sarker IH (2022). AI-based modeling: techniques, applications and research issues towards automation, intelligent and smart systems. SN COMPUT SCI..

[B061] Sasloglou KA, Glover I, Goh HG, Kwong KH, Gilroy MP, Tachtatzis C (2009). Antenna and Base-Station Diversity for WSN Livestock Monitoring. WSN.

[B062] Schori F, Münger A (2022). Assessment of two wireless reticulo-rumen pH sensors for dairy cows..

[B063] Selli A, Ventura RV, Fonseca PAS, Buzanskas ME, Andrietta LT, Balieiro JCC, Brito LF (2021). Detection and visualization of heterozygosity-rich regions and runs of homozygosity in worldwide sheep populations. Animals (Basel).

[B064] Seminger D, terHeerdt J, Brenner L, Duncan O, Sparkman M, Sherer T (2023). Data Types in power BI Desktop - Power BI.

[B065] Slob N, Catal C, Kassahun A (2021). Application of machine learning to improve dairy farm management: a systematic literature review. Prev Vet Med.

[B066] Souza AM, Weigert RDAS, Machado De Sousa EP, Tassoni Andrietta L, Ventura RV (2022). Practical implications of using non‐relational databases to store large genomic data files and novel phenotypes. J Anim Breed Genet.

[B067] Tullo E, Finzi A, Guarino M (2019). Review: environmental impact of livestock farming and Precision Livestock Farming as a mitigation strategy. Sci Total Environ.

[B068] Wang Y, Kang X, He Z, Feng Y, Liu G (2022). Accurate detection of dairy cow mastitis with deep learning technology: a new and comprehensive detection method based on infrared thermal images. Animal.

[B069] Watanabe RN, Bernardes PA, Romanzini EP, Braga LG, Brito TR, Teobaldo RW, Reis RA, Munari DP (2021). Strategy to predict high and low frequency behaviors using triaxial accelerometers in grazing of beef cattle. Animals (Basel).

[B070] Wu D, Han M, Song H, Song L, Duan Y (2023). Monitoring the respiratory behavior of multiple cows based on computer vision and deep learning. J Dairy Sci.

[B071] Wu D, Yin X, Jiang B, Jiang M, Li Z, Song H (2020). Detection of the respiratory rate of standing cows by combining the Deeplab V3+ semantic segmentation model with the phase-based video magnification algorithm. Biosyst Eng.

[B072] Zhang L, Kim J, Lee Y (2018). The platform development of a real-time momentum data collection system for livestock in wide grazing land. Electronics (Basel).

[B073] Zhao K, Bewley JM, He D, Jin X (2018). Automatic lameness detection in dairy cattle based on leg swing analysis with an image processing technique. Comput Electron Agric.

[B074] Zuidhof MJ, Afrouziyeh M, Chang-Fung-Martel J, You J, van der Klein SAS, Kyriazakis I (2023). Smart livestock nutrition..

